# Exploring Epigenetic Age in Response to Intensive Relaxing Training: A Pilot Study to Slow Down Biological Age

**DOI:** 10.3390/ijerph16173074

**Published:** 2019-08-23

**Authors:** Sofia Pavanello, Manuela Campisi, Francesco Tona, Carlo Dal Lin, Sabino Iliceto

**Affiliations:** 1 Occupational Medicine, Department of Cardiac, Thoracic, and Vascular Sciences and Public Health, University Hospital of Padua, Via Giustiniani, 2, 35128 Padova, Italy; 2 Clinical Cardiology, Department of Cardiac, Thoracic, and Vascular Sciences and Public Health, University Hospital of Padua, Via Giustiniani, 2, 35128 Padova, Italy

**Keywords:** epigenetic age, DNA methylation age, relaxing training, telomere length, myocardial infarction patient, telomerase

## Abstract

DNA methylation (DNAm) is an emerging estimator of biological aging, i.e., the often-defined “epigenetic clock”, with a unique accuracy for chronological age estimation (DNAmAge). In this pilot longitudinal study, we examine the hypothesis that intensive relaxing training of 60 days in patients after myocardial infarction and in healthy subjects may influence leucocyte DNAmAge by turning back the epigenetic clock. Moreover, we compare DNAmAge with another mechanism of biological age, leucocyte telomere length (LTL) and telomerase. DNAmAge is reduced after training in healthy subjects (*p* = 0.053), but not in patients. LTL is preserved after intervention in healthy subjects, while it continues to decrease in patients (*p* = 0.051). The conventional negative correlation between LTL and chronological age becomes positive after training in both patients (*p* < 0.01) and healthy subjects (*p* < 0.05). In our subjects, DNAmAge is not associated with LTL. Our findings would suggest that intensive relaxing practices influence different aging molecular mechanisms, i.e., DNAmAge and LTL, with a rejuvenating effect. Our study reveals that DNAmAge may represent an accurate tool to measure the effectiveness of lifestyle-based interventions in the prevention of age-related diseases.

## 1. Background

DNA methylation is an emerging robust biomarker of biological aging. The advent of epigenome-wide high-throughput sequencing analyses has led to a successful identification of a large number of genomic sites strongly associated with age [1]. These discoveries have allowed the creation of an “epigenetic clock” with unprecedented accuracy for age estimation with an average error of only 3.6 years; this is defined as DNA methylation age (DNAmAge) [1,2,3]. The methylation-based biological age was mostly developed based on DNA extracted from blood as an easy available source [1,2,3,4]. Researchers have developed several multiple age-prediction models with various statistical methods to determine the age of a person based on the age-dependent methylation changes in certain CpG loci [1,2,5,6]. The number of CpG sites employed in developing these age prediction models fluctuates from a small number to the 100 s. An attempt has been made to increase the feasibility of age predictors with the utilization of as few loci as possible. Amongst the most robust DNAmAge predictors is the model proposed by Zbieć-Piekarska et al. [3] that was developed using data from 5 CpG sites. This method shows that DNAmAge highly correlates (r = 0.94) with chronological age with a mean deviation from the calendar age (4.5 years) analogous to that from Horvath et al. [1] and Hannum et al. [2] (r = 0.96 and r = 0.91), with 3.6 and 4.9 years considered as the reference methods. Zbieć-Piekarska et al. [3] developed the algorithm from a larger sample (n = 420) and then they validated it using a smaller one (n = 300), covering the entire adult life span.

The discrepancy between DNAmAge and chronological age (ΔDNAmAge) [7], defined as age acceleration, that provides information regarding the speed of epigenetic clock [7], is closely associated with age-related disorders, including cardiovascular disease [8,9] and mortality risk [10,11]. Environmental factors have been found to impact age acceleration and include economic hardship [12], dietary factors [13], pollution [14,15] and education [16]. Cumulative lifetime stress hormones are one of the factors described to accelerate epigenetic aging [17,18]. On the other hand, one study examines the beneficial impact of physical exercise [19] and long-term meditation on DNAmAge [20]; however, no longitudinal study has been performed.

Relaxing practices, including meditation and music listening, are novel and inexpensive interventions that have become a focus of scientific interest. The American Heart Association (AHA) recommends the potential benefits of relaxing techniques for primary and secondary prevention of cardiovascular disease [21]. Relaxing practices are able to counteract stress and stress-induced disorders, through the activation of specific brain areas, evoking the relaxation response (RR) [22]. The RR is the complement of the stress response. Millennia-old rituals arousing the RR comprise meditation, yoga and repetitive prayer. The RR reduces levels of stress hormones, inflammation and oxidative stress [23,24,25] that are molecular pathways involved in cellular aging processes [26]. Studies show that some forms of meditation that help to mitigate psychological stress have a favorable effect on telomeres by activating telomerase activity [27,28]. The potential effect of intensive meditation training on telomere length elongation is suggested by two studies on healthy subjects [29,30], while no effect has been observed in breast cancer survivors [31,32] and depressed patients [33]. However, it is unexplored whether relaxing experiences modulate the rate of the epigenetic aging.

In our previous work, the beneficial impact of relaxing training has been demonstrated in patients after myocardial infarction (MI) with respect to inflammatory genes, across a cascade of neuro-endocrine-immune (NEI) messengers, and on clinical recovery of endothelial function and the initial regression of carotid atherosclerosis [34].

In this work, within the framework of a pilot longitudinal study, with a before-and-after design, we examine the hypothesis that an intensive relaxing training (twice a day) for 60 days may influence leucocyte DNAmAge by turning back the epigenetic clock in patients after MI and in healthy subjects. To explore this hypothesis we applied the prediction model proposed by Zbieć-Piekarska et al. [3] that presents high prediction accuracy and makes easier the application of age predictors’ analysis. Moreover, we compare DNAmAge with another mechanism of biological age, leucocyte telomere length (LTL) and telomerase. The longitudinal assessment of age-related biomarkers is the appropriate tool to determine accelerated aging, since confounding factors can be controlled.

## 2. Materials and Methods

### 2.1. Study Subjects

Within the framework of a pilot longitudinal study (before and after), in which each subject is a control of him/herself, we enrolled consecutively from October to December 2015, n = 20 patients who were admitted to our Cardiology Wards for ST elevated (STEMI) or non-ST elevated myocardial infarction (NSTEMI) and also with carotid atherosclerosis, and n = 10 healthy control individuals age- and gender-matched as previously described [34]. Patients and healthy controls were all Caucasians. Subjects were asked to participate in relaxing training. Each subject signed a consent form giving his or her agreement to take part in the study. All subjects were instructed in relaxing practices as previously described [34]. In brief, the initial four days of training took place in our hospital and the rest of the relaxing sessions was carried out independently by the subjects at home for 20 min, 2 times a day. Subjects, from whom a sufficient quantity of DNA and RNA was available, were analysed to evaluate DNAmAge, LTL and telomerase. In the group of patients and healthy subjects, individuals were 14 and 6 with 3 (21%) and 2 (33%) females per group, respectively. In these groups, age was quite differently distributed. However, no significant difference was found among age, sex and body mass index (BMI), except for smoking habits. Patients, however, soon after MI and during relaxing training, stopped active smoking. Our institutional ethic committee (protocol number 3487/AO/15) authorized the study.

**Figure 1:**
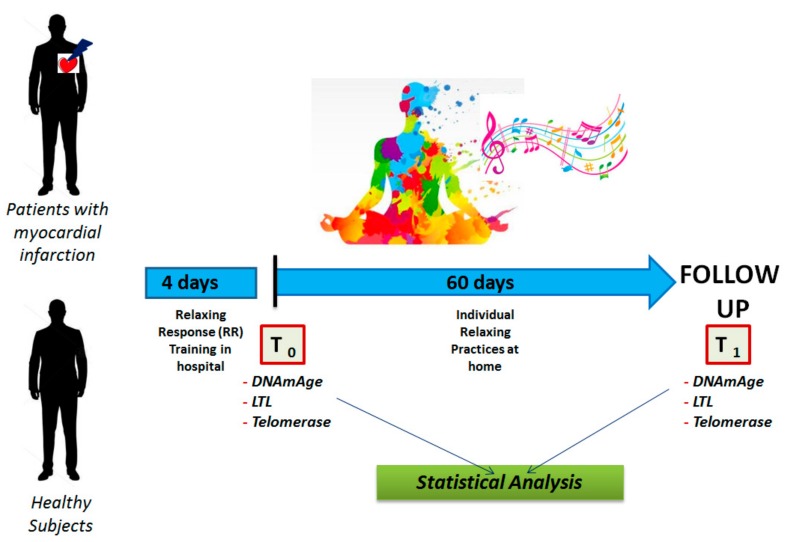
Longitudinal Plan of the Study.

In Figure 1, we describe the research plan. Briefly, at enrolment (T0) and after 60 days (T1) of relaxing practice, for each subject, blood samples were collected in order to analyse biological age DNAmAge and LTL from extracted DNA and telomerase from RNA. For each subject at T0 and T1, vital indicators were also measured and blood samples for biochemical parameters and stress hormones, inflammatory cytokines, oxidative stress markers, endothelial progenitor cells (EPCs) and the expression of inflammatory genes (p53, Nuclear Factor kappa-light-chain-enhancer of activated B cells, Toll-like receptor 4) were also taken [34]. An electrocardiogram and transthoracic echocardiography were completed in order to evaluate the arterial pressure as previously reported [34]. The environmental conditions at the time of data collection were the same for all the subjects. In particular, the coaching, the relaxing sessions and blood withdrawals were performed in the same laboratory of our clinic located near our echocardiography test centre. A treatment diary was kept by participants. Patients and controls were also evaluated by the Clinical Psychology Unit of our hospital to attest the individual personality characteristics, neuro-cognitive reserve and the grade of perceived stress. Briefly, all patients were free of cognitive deficit and had no other comorbidities. All patients received the best medical treatment in agreement with AHA and European Society of Cardiology recommendations for the therapy of ischemic heart disease and adhered to the same cardiac rehabilitation plan (physical exercise and nutrition training).

### 2.2. DNA Extraction

DNA was extracted from whole blood by the DNAeasy Blood&Tissue kit (QIAGEN, Milano, Italy) on a QIAcube System (QIAGEN, Milano, Italy) for automated DNA purification, following the manufacturer’s instructions according to a customized protocol. DNA was quantified and checked for quality using both Quantus™ Fluorometer (Promega, Milano, Italy) and QIAexpert Quantification System (QIAGEN, Milano, Italy). We obtained genetic material suitable for subsequent analytical procedures both from a qualitative (260/280 nm 1.8 for DNA) and quantitative (mean DNA recovery 98.6 ng/µL) point of view.

### 2.3. DNAmAge

DNAmAge was determined by analysis the methylation levels from selected markers using bisulfite conversion and Pyrosequencing^®^ methodology. This method is based on determination of the methylation level of a set of five markers (ELOVL2, C1orf132, KLF14, TRIM59 and FHL2) in genomic DNA, as described [3] with some modifications based on the fact that the method was completely automated using the PyroMark Q48 Autoprep (QIAGEN, Milano, Italy). Briefly, 2 μg DNA was submitted to bisulfite conversion: unmethylated cytosines in extracted DNA were converted to uracil using Epitect Fast^®^ DNA Bisulfite (QIAGEN, Milano, Italy) following the manufacturer’s instructions. An aliquot of template DNA was used for PCR amplification of selected markers using PCR primers included in the AgePlexMono kit (Biovectis, Warszawa, Poland). Details of sites and sequences for analysis are reported in Supplementary Materials Table S1. PCR reactions were performed in 25 μL, comprising 0.2 μM of each primers, 20 ng of template DNA, and PyroMark PCR Master Mix holding HotStarTaq DNA Polymerase, 1X PyroMark PCR Buffer and dNTPs. The amplification plan involved a preliminary denaturation step at 95 °C for 10 min, followed by 40–45 cycles of denaturation (94 °C for 30 s), annealing (54–56 °C for 60 s) and extension (72 °C for 90 s), and a final extension of 72 °C for 10 min. Each PCR amplification contained negative PCR controls. In total, 10 µL of PCR product was used for each pyrosequencing primer containing in AgePlex Mono kit (Biovectis) and loaded into a 48 well-plate (Pyromark Q48 Discs, QIAGEN, Milano, Italy). Pyrosequencing was performed on a Pyromark Q48 Autoprep instrument using Pyromark Q48 Advanced Reagents (QIAGEN) according to the manufacturer’s instructions. The resulting Pyrograms^®^ generated by the instrument were automatically analyzed using Pyromark Q48 Autoprep Software (QIAGEN, Milano, Italy). The level of methylation was expressed as a percentage of methylated cytosines at the 5 CpG sites considered. The methylation percentages were inserted in an online calculator system accessible at www.agecalculator.ies.krakow.pl, for estimation of biological age from DNA methylation analysis. The equation corresponds to a previously developed age prediction model [3]. All samples were analyzed 3 times for each marker to verify the reproducibility of our results, and their average was utilized in the statistical testing. All samples were analyzed on two different days, and the coefficient of variation (CV) for replicate pyrosequencing runs was 0.5%.

### 2.4. LTL and Telomerase Expression

LTL was measured by the real-time quantitative PCR method (qPCR) as previously described [35,36]. This analysis determines the TL in genomic DNA by establishing the ratio of telomere repeat copy number (T) to single-copy gene (S), T/S [35,36]. As a single-copy gene in this study, we used human β-globin (hbg). A “seven-points” standard curve was produced from a consecutively diluted DNA pool (obtained from DNA samples randomly chosen) in order to calculate the relative quantities of T and S (in nanograms). All samples and standards were examined in triplicate, and the mean of the 3 T/S ratio measures was employed in the statistical evaluations. The PCR runs were conducted in triplicate on a SteponePlus Real-Time PCR System (Life Sciences Solutions, Thermo Fisher Scientific, Monza, Italy). To test the reproducibility of measurements, samples were replicated on different days, and the CV for the average T/S ratio was 8.5%, which was similar to the CV previously reported [35,36]. The expression of hTERT was analyzed by real-time PCR using TaqMan Gene Expression assay (Life Sciences Solutions, Thermo Fisher Scientific, Monza, Italy).. hTERT expression was standardized on GAPDH expression. All the reactions were performed in 96-well plates by the Steponeplus Real Time PCR System. The profile of thermal cycles and the PCR reaction components for both cDNAs were those recommended by the Protocol TaqMan ^®^ Gene Expression Assays (Life Sciences Solutions, Thermo Fisher Scientific, Monza, Italy). The relative gene expression levels were determined on the basis of the ∆∆Ct method of Livak and Schmittgen [37]. The comparative cycle threshold method (∆∆Ct), which compares the difference in cycle threshold values between groups as previously described [37], was used to obtain the relative fold change in gene expression.

### 2.5. Sample Size Estimation

Sample size estimation for before-and-after study (Paired T-test) was applied to calculate the sample size for all subjects, patients and healthy subjects. For each group, the mean of the change Δ_T1 − T0_ DNAmAge and the relative standard deviation (SD) were calculated (see Table 1). The effect size, obtained using the T statistic and non-centrality parameter, was 2.33, 8.29 and 2.88 for patients, healthy subjects and all subjects, respectively. It was calculated by considering α (two-tailed) = 0.05, β = 0.20 and SD = 2.88, 5.78 and 4.36 for patients, healthy subjects and all subjects, respectively. The calculation of sample size was computed through a STATA command by specifying the sample size (14 patients, 6 healthy subjects and 20 all subjects), the SD and the effect size. The group size to obtain statistical significance with α (two-tailed) = 0.05 and β = 0.20 was estimated to be n = 18, 14 and 4, respectively, for all subjects, patients and healthy subjects.

### 2.6. Statistical Analysis

In the text, continuous variables are expressed as the mean and standard deviation, while dichotomous variables are expressed as percentages. DNAmAge and the difference between DNAmAge after and before the relaxing practices (Δ_T1 − T0_ DNAmAge) are expressed as the mean and standard deviation, such as LTL and telomerase expression. Statistical analysis was applied to compare DNAmAge and LTL before and after 60 days of relaxing practices and to analyse their relationship. Comparison between two groups was made using a (two-tailed) paired *t* test, and correlation between means was evaluated by non-parametric linear regression models (Spearman’s and Kendall’s ranks). Multiple linear regression analysis was performed to examine the influence of disease, gender, treatment, and chronological age (independent variables) on DNAmAge and LTL (dependent variable) of all study subjects. We used Chow’s test, a test to appraise whether the coefficients, of two linear regressions on different data sets, presented a similar trend. All statistical tests and *p*-values were two-sided and were conducted with Statsdirect Statistical software (Ashwell). Results were considered significant when a *p*-value of ≤0.05 was obtained.

## 3. Results

### 3.1. DNAmAge Correlation with Chronological Age

**Figure 2:**
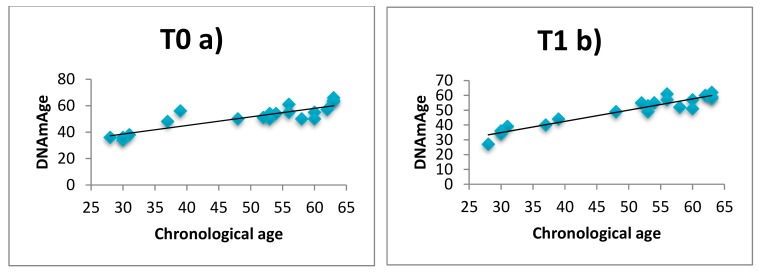
Correlation curves between DNAmAge and chronological age at enrolment T0 (**a**) versus after 60 days of relaxing practices T1 (**b**).

Linear regression analysis showed that in all subjects (n = 20) DNAmAge is high positively correlated with chronological age both before (T0) and after 60 days (T1) as displayed in Figure 2a/Figure 2b (Kendall’s rank correlation, a) tau = 0.78, *p* < 0.01; b) tau = 0.89, *p* < 0.0001), with a mean deviations from calendar age of 4.36 and 1.3 years. This result is a quality validation of our analysis, confirming the extreme accuracy for epigenetic age estimation of this model. The multiple linear regression results (data not shown) confirmed that DNAmAge is highly dependent both before and after intervention (analysis of variance F = 21.01 *p* < 0.0001 and F = 54.06 *p* < 0.0001) on chronological age (r = 0.758, *p* < 0.001 and r = 0.719, *p* < 0.001) but not from gender (r = 0.045, *p* = 0.859 and r = −0.409, *p* = 0.092) and from to be patients (r = −0.446, *p* = 0.063 and r = 0,137, *p* = 0.587).

### 3.2. DNAmAge after 60 Days of Relaxing Practices

DNAmAge and Δ_T1 − T0_ DNAmAge of all subjects, patients and healthy subjects, before and after intervention, are shown in Table 1. DNAmAge of healthy subjects after 60 days (T1) of relaxing practices is significantly younger (Δ_T1 − T0_ DNAmAge = −4.66 years, *p* = 0.053), but not that of patients (Δ_T1 − T0_ DNAmAge = −0.14 years; *p* = 0.428). This indicates a decrease in DNAmAge after relaxing practices in healthy subjects but not in patients. Multiple linear regression results (Table 2) confirm that Δ_T1 − T0_ DNAmAge decrease/ rejuvenation is highly dependent on healthy subjects (r = 0.631, *p* = 0.005) and declines with chronological age (r = −0.507, *p* = 0.032), but not gender (r = −0.443, *p* = 0.075).

Table 3 shows DNA methylation status at the CpG sites of each of the five genes analyzed for DNAmAge determination (ELOVL2, C1orf132, KLF14, TRIM59 and FHL2). We find a significant decrease in DNA methylation pattern of KLF14 in all subjects after intervention (T1 versus T0 mean KLF14 % methylation (met) = 11.5 versus 13.3; Paired *t* test = 2.23; *p* = 0.037) suggesting that this gene is more susceptible to epigenetic changing.

### 3.3. LTL, Telomerase and Relaxing Practices

**Figure 3:**
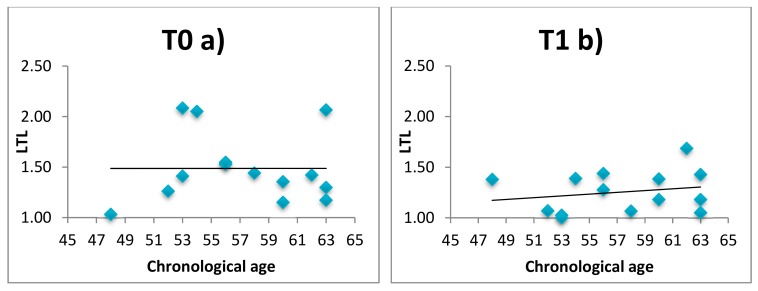
Comparison of the correlation curves between LTL and chronological age at enrolment T0 (**a**) versus after 60 days T1 (**b**) based on Chow’s test for patients. Chow Test F (2, 24 df) = 73,975 (*p* < 0.01).

**Figure 4:**
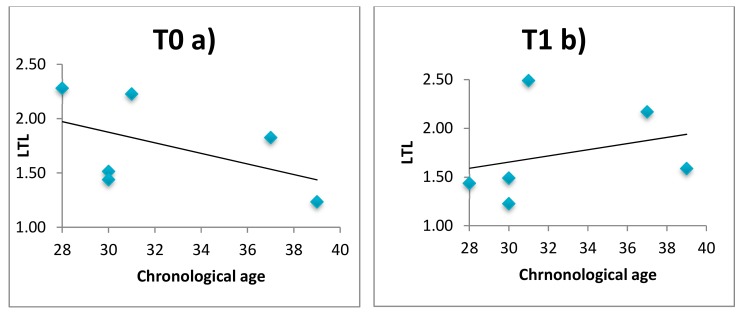
Comparison of the correlation curves between LTL and chronological age at enrolment T0 (**a**) versus after 60 days T1 (**b**) based on Chow’s test for healthy subjects. Chow Test F (2, 8 df) = 11,889 (*p* < 0.05).

LTL and telomerase were evaluated in 14 patients and 6 healthy subjects before (T0) and after intervention (T1). Results of LTL and telomerase expression are reported in Supplementary Materials Table S2. After 60 days of intervention LTL is preserved in healthy subjects, while is continues to decrease in patients (T1 versus T0 mean LTL = 1.25 versus 1.49, *p* = 0.05). Telomerase expression did not differ in all groups. Moreover, we observed that the conventional negative correlation between LTL and chronological age is negative at enrolment (T0) but becomes significantly positive at T1, i.e., after 60 days of relaxing practices, in both patients (Figure 3) and healthy subjects (Figure 4). Comparison of the correlation curves by Chow’s test at T0 versus T1 shows a significant difference in patients and healthy subjects (Chow Test F (2, 24 df) = 7.40 (*p* < 0.01); Chow Test F (2, 8 df) = 1.20 (*p* < 0.05)]. Correlation between DNAmAge and LTL was not significant in patients (T0 tau b = 0.012, *p* > 0.999; T1 tau b = 0.268, *p* = 0.206) and healthy subjects (T0 tau b = −0.276, *p* = 0.566; T1 tau *b* = 0.467; *p* = 0.259) both before and after intervention (data not shown).

## 4. Discussion

In this longitudinal study, we explored the hypothesis that intensive (twice a day) relaxing training may influence leucocyte DNAmAge, turning back the epigenetic clock, in patients after myocardial infarction, and in healthy subjects, using the model proposed by Zbieć-Piekarska et al. [3]. This method has high prediction accuracy and make easier the application of age predictor analysis. Moreover, we compared DNAmAge with LTL and telomerase, other indicators of biological aging.

We found a reduction in DNAmAge in healthy subjects after 60 days of intensive relaxing training, but not in patients. The multiple linear regression results confirm that DNAmAge rejuvenation is highly dependent on healthy subjects but decreases with chronological age. Our results are in line with those obtained by Chaix and co-workers [20], who investigated in a cross-sectional study the effects of regular meditation practices on DNAmAge in healthy long-term meditators. They found a beneficial effect on DNAmAge in relation with several years of meditation. The results of our longitudinal study add that meditation practices have a potential rejuvenating effect on DNAmAge even after 60 days of relaxing practices in healthy subjects but are not sufficient in patients after myocardial infarction and in elderly subjects. One plausible mechanism that may support the positive effect of relaxing practices is the epigenetic switching through stress hormones. Stressors can drive a stable alteration in DNA methylation [17,18,38]; this change is mediated by the genomic action of glucocorticoids, primary molecular effectors to stress response [17,18]. Glucocorticoids act in every bodily organ, by stimulation of a transcription factor, the glucocorticoid receptor (GR) that regulates gene expression with the binding of its homodimer to glucocorticoid response elements (GREs) in regulatory regions [39]. Beyond regulating gene transcription, GRE binding can locally produce demethylation, a form of molecular signature that influences ensuing responses to glucocorticoids [40]. In both groups (patients and healthy subjects), we observed a significant reduction of stress hormones (cortisol, ACTH, copeptin, epinephrine and norepinephrine) after relaxing training [34]. Therefore, it is plausible that stress hormone reduction in our healthy subjects throughout the relaxing practices could influence cellular aging through DNA demethylation. The null findings for DNAmAge in the intervention group of patients could be explained by the fact that stress hormone disruption is higher in patients than in healthy subjects. In our patients, for example, at time T0 we observed that epinephrine and norepinephrine were higher than in healthy subjects [34]. This would suggest that more time and therefore a longer period of relaxing therapy is necessary to reduce hormone levels in patients than in healthy subjects. The fact that the rejuvenation effect decreases with age is in agreement with the fact that elderly people are more sensitive to stress and epigenetic changing mediated by glucocorticoids, due to the decline in DNA repair and maintenance mechanisms [41].

Moreover, we found a significant decrease in the DNA methylation pattern of KLF14 after intervention. KLF14 is part of the Krüppel-like factor family, which is a zinc-finger family of transcription factors capable of linking to GC-rich sequences, and has appeared as a key controller of important functions in various organs [42]. It especially regulates transcription cytokines and markers of inflammation [43] and appears to be associated with coronary artery disease [44]. Among the ageing-associated CpG sites, the KLF14 promoter region has been related to chronological age in several populations with distinctive ethnic backgrounds [45,46]. Therefore, the reduction detected in the methylation pattern of KLF14 after relaxing practices could provide a new indicator of the favorable effect, in combination with the decrease in cytokines and markers of inflammation (ESR, fibrinogen, HS-CRP, IL-6 and TGFβ-1) we found [34].

The methylation-based biological age is an attractive biomarker of aging because it can be measured in the DNA of most human tissues with high accuracy [1,4]. We chose to use the model proposed by Zbieć-Piekarska et al. [3] to increase the practicability of the test by detecting methylation levels of only 5 genes: ELOVL2, FHL2, KLF14, TRIM59 and C1orf132. Specifically in the first four genes, methylation is fostered with age, while in the latter one, methylation declines with age [3]. In this work, DNAmAge was highly correlated with chronological age in all subjects, confirming the prediction power of this model with a mean deviation from calendar age very comparable to those of Horvath [1] and Hannum et al. [2] and the quality validation of our analysis.

We found that after relaxing practices, LTL is preserved in healthy subjects, but continues to decrease in patients. Furthermore, we found that the conventional negative correlation between LTL and chronological age becomes positive after training both in patients and in healthy controls. This result suggests that relaxing training may have a positive impact on LTL regulation, both in healthy subjects and patients. Our results agree with those reporting in prospective studies a beneficial influence of meditation practices on LTL in healthy subjects, specifically after 3 weeks [30] and 12 weeks intervention [29], and in breast cancer survivors in whom LTL was instead maintained after 8 weeks of mindfulness practices [31].

Moreover, we did not find any differences in telomerase expression after relaxing practices. Our results are in accordance with those observed in non-regular meditators after 3 weeks [30] and after 6 days [47] of a meditation retreat or relaxing practices. However, other studies found an increase in telomerase activity in healthy subjects [27,48] and in breast cancer survivors [32]. The heterogeneity of telomerase activity findings makes it tricky to discriminate a clear relationship between meditation practice and telomerase across studies. As observed by Conklin and co-workers [30] the increase in LTL may result from both telomerase-mediated and telomerase-independent pathways.

No correlation between DNAmAge and LTL was found in all subjects, both before and after intervention. This result is in line with recent studies [49,50,51] that did not show LTL to be associated with epigenetic age. These biological age indicators respond to molecular mechanisms completely differently.

qPCR is the most frequently applied method for LTL testing even if crucial phases of analysis may contribute to the assay variability [52]. These include preanalytical factors, such as DNA extraction methods which are the most important sources of experiment-induced variation in qPCR LTL analyses, and analytical factors, i.e., telomere primer sequences and concentration, single copy gene, master mix, PCR program conditions, inclusion of quality control samples, PCR instruments and data analysis method. In this longitudinal study, the DNA was extracted from whole blood samples with the same DNA extraction kit. Moreover, analytical factors did not differ among analyses, and the same pool DNA was used as a reference standard in each qPCR reaction. All together, these procedures make possible to decrease the assay variability, reducing the measurement error.

The weakness of our study is mainly related to the low number of subjects, in particular in the healthy group; however, the sample size estimation we calculated is sufficient to obtain statistical significant results. Another weakness of the study could be the imbalanced ratio of males to females (i.e., 3 in patients and 2 in healthy subjects group), smokers to nonsmokers and the different distribution of age. This is a longitudinal study in which each subject was studied twice, before-and-after 60 days of relaxing practices; hence, each subject is the control of him/herself with the second observation reinforcing the first. Moreover, the multiple regression analyses do not show any influence of gender on DNAmAge and LTL, neither in patients nor in healthy subjects, both before and after relaxing practices. Concerning smoking, a previous study failed to detect an effect of smoking on DNAmAge in blood [13]; several studies, including large investigations such as that conducted by Bischoff et al. [53] and Cassidy et al. [54], were unable to confirm the negative correlation between LTL and smoking found by others [55]. Furthermore, patients soon after MI and during relaxing training stopped active smoking. The inconsistency between several studies likely seems to reflect a complex effect of smoking, if any, on LTL that might not be easily detectable. Conversely, the association of an overall impact of smoking on DNAmAge is still unexplored and deserves further study.

The longitudinal characteristic is one strength of the present research, indeed all subjects (patients and healthy subjects) were analyzed before and after 60 days of relaxing practices and each subject is the control of him/herself in a short-term period. The before-and-after design offers better evidence about intervention effectiveness than a cross-sectional study and is most useful in demonstrating the impacts of a short-term program. To minimize the threats of validity, several precautions were taken for our short-term study. These include the scrupulous timing of blood sampling, the environmental conditions at the time of data collection; in particular, the coaching, the relaxing sessions and blood withdrawals were performed for all subjects in the same laboratory of our clinic located near our echocardiography test center; a treatment diary was kept by each participant; patients and healthy subjects were also evaluated by the Clinical Psychology Unit to attest to any individual personality characteristics; assessment of parameters specifically connected to RR (including stress hormones, inflammatory genes, across a cascade of neuro-endocrine-immune (NEI) messengers, and on clinically regaining of endothelial function and the initial regression of carotid atherosclerosis), whose changes can be ascribed to relaxing practices according to the methods used, as reported in our previous work [34]. All these measures were taken in order to preclude any other influences. Moreover, our data are in line with the biological assumption that relaxing practices by eliciting RR may influence leucocyte DNAmAge by turning back the epigenetic clock. Taking into account the above and the longitudinal nature of the study, we can assume that sample size, individual and technical conditions did not affect our findings, and the differences we observed before and after relaxing practices are related to treatment.

The innovation introduced by this study consists not only of the simultaneous evaluation of two indicators of biological aging but also of the perspective nature of the study design in which each subject was a self-control. In this way, it is possible to overlook the influence of previous events that might have contributed to modify indicators of biological aging and the other functional, vital, genetic and biochemical parameters we detected.

## 5. Conclusions

The new relevant finding stemming from our study is a potential decrease of DNAmAGE after 60 days of intensive relaxing practices especially in healthy subjects, suggesting a rejuvenating effect of these practices. We also observed a reduction in the methylation pattern of KLF14 after relaxing practices that may provide a new indicator of the favorable effect on prevention coronary artery disease. The analysis of the epigenetic clock therefore may represent an accurate tool to measure the effectiveness of lifestyle-based interventions for the prevention of age-related diseases. Moreover, the conventional negative correlation between LTL and chronological age that became positive after relaxing training suggests that these techniques may have a positive influence on telomere regulation. Our findings, on epigenetic age, corroborated by those on LTL, support our hypothesis of the benefits of intensive relaxing practices, which influence two key molecular mechanisms involved in cellular aging, and could represent a novel and inexpensive preventive strategy for stress- and age-related chronic diseases. However, we recognize the fact that the low number of people analyzed in this study raises the question of chance findings. Longitudinal studies in larger cohorts are therefore required to validate and further characterize these findings.

## Figures and Tables

**Table 1:** DNAmAge and Δ_T1 − T0_ DNAmAge at enrolment T0 and after 60 days of relaxing practices T1.

	T0	T1		*p ^§^*
	DNAmAge		Δ_T1 − T0_ DNAmAge	
	Mean (SD)			
All subjects	51.4 (9.37)	49.9 (10.0)	1.50 (4.36)	0.143
Patients	55.7 (5.66)	55.6 (4.29)	−0.14 (2.88)	0.428
Healthy subjects	41.3 (8.73)	36.7 (5.85)	−4.67 (5.78)	**0.053**

*^§^* Paired two sided *t* tests.

**Table 2:** Multiple linear regression of the influence of being healthy subject, age and gender on Δ_T1 − T0_ DNAmAge for all subjects (n = 20).

	b	r	t	*p*
Healthy subjects	14.836	0.631	3.260	**0.005**
Chronological age	−0.400	−0.507	2.350	**0.032**
Gender	−3.497	−0.443	1.977	0.075

Analysis of variance from regression: F = 5.297 *p* = 0.01.

**Table 3:** Methylation levels (% met) of five selected markers at enrolment T0 and after 60 days of relaxing practices T1.

	T0	T1	*p^§^*
	ELOVL2 % Met Mean (SD)		
All subjects	61.2 (4.46)	61.9 (6.02)	0.253
Patients	63.0 (2.66)	64.6 (3.86)	0.071
Healthy subjects	56.8 (5.04)	55.7 (5.61)	0.135
	**C1orf132 % Met mean (SD)**		
All subjects	49.7 (10.9)	40.9 (8.52)	0.554
Patients	45.4 (9.35)	46.9 (6.55)	0.517
Healthy subjects	59.8 (6.88)	60.2 (3.97)	0.935
	**TRIM59 % Met mean (SD)**		
All subjects	50.6 (6.57)	51.9 (7.67)	0.204
Patients	53.9 (4.26)	56.1 (3.61)	0.131
Healthy subjects	43.2 (4.62)	42.2 (5.04)	0.110
	**KLF14 % Met mean (SD)**		
All subjects	13.3 (3.21)	11.5 (1.99)	**0.037**
Patients	13.4 (2.95)	12.5 (1.45)	0.260
Healthy subjects	12.8 (4.02)	9.2 (0.41)	0.087
	**FHL2 % Met mean (SD)**		
All subjects	45.0 (8.09)	45.7 (6.88)	0.609
Patients	47.7 (8.06)	48.6 (6.06)	0.671
Healthy subjects	38.5 (2.95)	39.0 (2.76)	0.774

*^§^* Paired *t* tests.

## Supplementary Materials

The following are available online at https://www.mdpi.com/1660-4601/16/17/3074/s1, Table S1: Details of markers, AgePlex sequences to analyze, CpG sites and location of the prediction model. Table S2: LTL and Telomerase expression at enrolment T0 and after 60 days of relaxing practices T1.
