# Supplementary material for: Exploring Epigenetic Age in Response to Intensive Relaxing Training: A Pilot Study to Slow Down Biological Age
Source: Int J Environ Res Public Health. 2019 Aug 23;16(17):3074. doi: 10.3390/ijerph16173074 (PMC6747190; doi:10.3390/ijerph16173074)
Supplement: Supplementary file 1 [file ijerph-16-03074-s001.pdf]

Supplementary Material

Table S1. Details of markers, AgePlex sequences to analyze, CpG sites and location of the prediction model.

marker	Sequence to analyze	CpG site	Location (GRCh38)
**ELOVL2**	CCRTAAACRTTAAACCRCCRCRCRAAACCRAC	C7	Chr6: 11044661
**C1orf132**	AAATCTACRCAAACRACRATAAATAATCC	C1	Chr1: 207823681
**TRIM59**	GGTTTGGYGYGGGAYGAGGYGAAGYGTYGG TGGTYGAYGGTTTTTGAGGAATTATTTTTTATTT	C7	Chr3: 160450199
**KLF14**	TYGYGTTTTTTTTTTTGTYGGYGAGTTAGGTA ATGGTAATAGAG	C1	Chr7: 130734355
**FHL2**	AGTTATYGGGAGYGTYGTTTTYGGYGTGGG TTTTYGGGYGYGAGTTTYGGAYGAGGTTTGGG	C2	Chr2: 105399288

Table S2. LTL and Telomerase expression at enrolment T0 and after 60 days of relaxing practices T1.

	**T0**	**T1**	$p^{\S}$
	**LTL** ***mean (SD)***		
**All subjects**	1.57 (0.38)	1.40 (0.38)	0.081
**Patients**	1.49 (0.34)	1.25 (0.20)	**0.051**
**Healthy subjects**	1.75 (0.43)	1.73 (0.49)	0.926
	**Telomerase** ***mean (SD)***		
**All subjects**	1.26 (0.86)	1.16 (0.57)	0.537
**Patients**	1.43 (0.89)	1.19 (0.66)	0.224
**Healthy subjects**	0.85 (0.66)	1.09 (0.31)	0.339

§Paired t tests
